# Supplementary material for: Lipidomics and Transcriptome Reveal the Effects of Feeding Systems on Fatty Acids in Yak’s Meat
Source: Foods. 2022 Aug 26;11(17):2582. doi: 10.3390/foods11172582 (PMC9455248; doi:10.3390/foods11172582)
Supplement: Supplementary file 1 [file foods-11-02582-s001.zip › foods-1851350-supplementary.htm]

MDPI login


### Login

Forgot your password?

Not registered yet?
Register now.

Login with your ORCID iD

This login page provides you access to the following platforms operated by MDPI:


© 1996-2022 MDPI (Basel, Switzerland) unless otherwise stated
